# Predicting anthropometric and metabolic traits with a genetic risk score for obesity in a sample of Pakistanis

**DOI:** 10.1038/s41598-021-87702-0

**Published:** 2021-04-15

**Authors:** Sobia Rana, Adil Anwar Bhatti

**Affiliations:** Molecular Biology and Human Genetics Laboratory, Dr. Panjwani Center for Molecular Medicine and Drug Research (PCMD), International Center for Chemical and Biological Sciences (ICCBS), University of Karachi, Karachi, 75270 Pakistan

**Keywords:** Genetics, Molecular biology

## Abstract

Obesity is an outcome of multiple factors including environmental and genetic influences. Common obesity is a polygenic trait indicating that multiple genetic variants act synergistically to influence its expression. We constructed a genetic risk score (GRS) based on five genetic variants (*MC4R* rs17782313, *BDNF* rs6265, *FTO* rs1421085, *TMEM18* rs7561317, and *NEGR1* rs2815752) and examined its association with obesity-related traits in a sample of Pakistanis. The study involved 306 overweight/obese (OW/OB) and 300 normal-weight (NW) individuals. The age range of the study participants was 12–63 years. All anthropometric and metabolic parameters were measured for each participant via standard procedures and biochemical assays, respectively. The genetic variants were genotyped by allelic discrimination assays. The age- and gender-adjusted associations between the GRS and obesity-related anthropometric and metabolic measures were determined using linear regression analyses. The results showed that OW/OB individuals had significantly higher mean ranks of GRS than NW individuals. Moreover, a significant association of the GRS with obesity-related anthropometric traits was seen. However, the GRS did not appear to affect any obesity-related metabolic parameter. In conclusion, our findings indicate the combined effect of multiple genetic variants on the obesity-related anthropometric phenotypes in Pakistanis.

## Introduction

Obesity is a multifactorial and complex metabolic disorder involving a chronic imbalance of energy homeostasis that has adverse implications for health such as dyslipidemia, arterial hypertension, coronary heart disease, type 2 diabetes mellitus, ovarian polycystosis, gallbladder lithiasis, sleep apnea syndrome, arthropathy, cerebral vasculopathy, and some neoplasms^1^. The substantially increasing worldwide prevalence of obesity has made it one of the major global public health concerns^2^. It is generally ascribed to unhealthy lifestyle choices or environmental factors but it is also known to have a strong genetic component. Thus, obesity is highly heritable and inherent genetic variations can confer augmented predisposition in some people while protection in others^3, 4^. In rare instances, genetic predisposition to obesity can be owing to a large-effect mutation that disrupts energy homeostasis^5^. Nevertheless, no such monogenic mutation can be indicated for the prevailing majority of severely obese people^6–8^. The genetic predisposition to obesity is but rather an outcome of the cumulative effects of several genetic variants with individually modest effects. This polygenic inheritance involving numerous common genetic variants has been reported to account for the majority of inherited predisposition to complex and common diseases in addition to obesity^9–11^. Therefore, it can be said that a single genetic variant can significantly affect the risk of disease occurrence in case of monogenic Mendelian disorders while multiple genetic variants (that individually play only a modest role in conferring disease risk) collectively make a considerable contribution in manifestation of complex traits and common disorders. In this context, the concept of genetic risk score (GRS) has emerged by which the cumulative small effect sizes of multiple genetic variants can be exploited to generate an overall score for predicting these traits and disorders. The GRS has been shown to provide reliable risk prediction for many complex genetic traits^12^. In particular, the remarkable potential of GRS for identifying individuals at a higher risk of developing obesity has been revealed^13, 14^.

The GRS is based on simple counts of disease-causing alleles. Generally, the GRS of any complex genetic trait can be computed by adding up risk alleles. Calculating a GRS by uniting information for multiple genetic variants offers a tool for examining genetic contributions in the manifestation of obesity in samples much smaller than are required for GWAS (genome-wide association studies)^15, 16^. Most GRS studies on obesity have been conducted on western populations so far^14, 17^. Thus at present, extending the scope of genetic studies to examine populations of other descents or under-represented populations is warranted^18^. Pakistani population is an under-represented population in this context. Pakistan is a South Asian country and its population offers many advantages in revealing the obesity etiology, which are attributable to its distinctive features including its recent nutritional transition, complex demographic history, diversity, endogamy, and consanguineous marriage practices resulting in a high rate of inbreeding, and the presence of large families^19^. Moreover, Pakistan has been recently ranked among the top 10 most obese countries in the world as per the Global Burden of Disease Study 2013^20^. Therefore, the current study has been undertaken to compute the GRS based on five obesity-linked key loci including *MC4R* rs17782313, *BDNF* rs6265, *FTO* rs1421085, *TMEM18* rs7561317, and *NEGR1* rs2815752 in this at-risk and under-represented population. The aforementioned genetic variants were previously reported to be individually associated with overweight/obesity in large GWASs on western populations^21, 22^ as well as in a sample of Pakistani population^23–26^.

The *FTO* (fat mass and obesity-associated) gene is the first obesity-susceptibility locus identified by GWAS^27, 28^. The upregulation of *FTO* leads to increased fat mass and obesity possibly through hyperphagia and/or low energy expenditure^29, 30^. MC4R (melanocortin 4 receptor) and BDNF (brain-derived neurotrophic factor) play a crucial role in the central regulation of energy homeostasis via induction of satiety and increased energy expenditure^31, 32^. TMEM18 (transmembrane protein 18) has also been indicated to play a role in the central regulation of appetite and energy expenditure as germline loss of *TMEM18* in mice resulted in increased body weight driven by increased food intake and aggravated by high-fat diet while selective overexpression of *TMEM18* in the paraventricular nucleus of wild-type mice decreased food intake and also enhanced energy expenditure^33^. NEGR1 may affect body weight via influencing energy balance, fat production and transport, and the structure of brain areas involved in feeding behavior^34–37^. Thus, risk variants within or near the aforementioned genes, which either increase or decrease their expression and/or function can result in insatiable appetite and/or low energy expenditure; consequently, leading to weight gain and obesity.

Previously, we sought individual associations of the aforementioned five genetic variants with overweight/obesity and related anthropometric and metabolic traits whereas now in the current study we seek the association of the GRS based on all the aforementioned five genetic variants with the obesity-related anthropometric as well as metabolic measures in the same sample of Pakistani population.

## Results

### Sample description

The ages of the sample population ranged from 12 to 63 years. The total sample population was composed of 606 individuals including 270 females and 336 males. There were an equal number of males (n = 168) in both overweight/obese (OW/OB) and normal-weight (NW) groups. However, there were 138 and 132 females in OW/OB and NW groups, respectively.

### Significant differences in study variables including GRS between OW/OB and NW Individuals

First, all anthropometric and metabolic parameters were compared between OW/OB and NW individuals. Anthropometric parameters included body weight, height, body mass index (BMI), waist circumference (WC), hip circumference (HC), skinfold thicknesses (SFTs), waist-to-hip ratio (WHR), waist-to-height ratio (WHtR), body fat percentage (%BF). Similarly, metabolic parameters encompassed systolic blood pressure (SBP), diastolic blood pressure (DBP), the product of triglycerides and glucose (TyG), fasting blood glucose (FBG), fasting insulin, homeostatic model assessment-insulin resistance (HOMA-IR), total cholesterol (TC), triglyceride (TG), high-density lipoprotein cholesterol (HDL-C), low-density lipoprotein cholesterol (LDL-C), very-low-density-lipoprotein cholesterol (VLDL-C), coronary risk index (CRI), atherogenic index (AI), triglyceride-to-HDL-C ratio (TG/HDL-C). All anthropometric and metabolic parameters except height, HDL-C, and CRI differed significantly between OW/OB and NW individuals being higher in OW/OB as compared with NW individuals (Table 1). Similarly, the mean ranks of GRS were found to differ significantly between OW/OB and NW individuals being higher in OW/OB as compared with NW individuals (Table 2).

**Table 1:** Comparison of the obesity-related anthropometric and metabolic parameters between OW/OB and NW individuals.

Parameters (unit of measurement)	Overweight/obese individuals		Normal-weight individuals		*p* value	*q-*value
	Mean ± SD	Mean ranks	Mean ± SD	Mean ranks		
Weight (Kg)	89.01 ± 19.25	439.83	59.20 ± 7.91	164.45	< 0.001	**< 0.001**
Height (cm)	164.27 ± 9.36	296.39	164.10 ± 8.55	310.76	0.312	0.312
BMI (Kg/m^2^)	32.89 ± 6.07	453.44	21.70 ± 2.01	150.56	< 0.001	**< 0.001**
WC (cm)	110.73 ± 14.26	444.08	82.95 ± 8.24	160.11	< 0.001	**< 0.001**
HC (cm)	114.61 ± 12.02	440.34	94.89 ± 5.80	163.92	< 0.001	**< 0.001**
WHR	0.97 ± 0.06	405.91	0.87 ± 0.07	199.04	< 0.001	**< 0.001**
WHtR	0.99 ± 0.33	442.78	0.50 ± 0.05	161.43	< 0.001	**< 0.001**
Biceps SFT (mm)	19.21 ± 8.74	410.28	8.89 ± 4.94	194.58	< 0.001	**< 0.001**
Triceps SFT (mm)	31.36 ± 9.94	430.44	14.89 ± 6.74	174.02	< 0.001	**< 0.001**
Sub-scapular SFT (mm)	33.20 ± 11.18	425.03	17.26 ± 6.18	178.12	< 0.001	**< 0.001**
Abdominal SFT (mm)	47.26 ± 15.40	428.19	24.28 ± 9.36	176.32	< 0.001	**< 0.001**
Thigh SFT (mm)	47.11 ± 16.72	431.91	20.41 ± 9.10	172.52	< 0.001	**< 0.001**
Supra-iliac SFT (mm)	38.65 ± 14.83	422.04	19.13 ± 8.07	182.59	< 0.001	**< 0.001**
% BF	34.94 ± 6.19	437.13	20.34 ± 6.97	167.20	< 0.001	**< 0.001**
SBP **(**mmHg)	119.90 ± 14.93	340.85	113.40 ± 13.46	265.40	< 0.001	**< 0.001**
DBP **(**mmHg)	79.80 ± 11.05	342.52	74.51 ± 9.52	263.70	< 0.001	**< 0.001**
TyG index	8.74 ± 0.51	357.78	8.44 ± .49	248.14	< 0.001	**< 0.001**
FBG (mg/dL)	105.10 ± 24.50	329.17	99.51 ± 12.55	277.32	< 0.001	**< 0.001**
Fasting insulin (µl U/mL)	27.01 ± 14.49	366.18	18.83 ± 12.21	239.57	< 0.001	**< 0.001**
HOMA-IR	6.99 ± 3.95	368.39	4.76 ± 3.97	237.31	< 0.001	**< 0.001**
TC (mg/dL)	157.79 ± 39.26	328.59	145.87 ± 38.85	277.91	< 0.001	**< 0.001**
TG (mg/dL)	135.55 ± 75.42	353.42	104.48 ± 57.78	252.58	< 0.001	**< 0.001**
HDL-C (mg/dL)	30.974 ± 9.39	313.36	29.82 ± 9.17	293.45	0.161	0.161
LDL-C (mg/dL)	103.42 ± 37.09	362.64	79.51 ± 31.48	243.17	< 0.001	**< 0.001**
LDL-C (mg/dL)	27.00 ± 15.04	349.74	21.18 ± 11.41	255.16	< 0.001	**< 0.001**
CRI	5.46 ± 1.91	314.69	5.15 ± 1.52	292.08	0.122	0.131
AI	3.47 ± 1.26	351.10	2.81 ± 1.14	254.95	< 0.001	**< 0.001**
TG/HDL-C	4.72 ± 3.14	341.24	3.88 ± 2.98	265.00	< 0.001	**< 0.001**

*OW/OB* overweight/obese, *NW* normal-weight, *BMI* body mass index, *WC* waist circumference, *HC* hip circumference, *WHR* waist-to-hip ratio, *WHtR* waist-to-height ratio, *SFT* skinfold thickness, *% BF* body fat percentage, *SBP* systolic blood pressure, *DBP* diastolic blood pressure, *TyG* the product of triglycerides and glucose, *FBG* fasting blood glucose, *HOMA-IR* homeostatic model assessment-insulin resistance, *TC* total cholesterol, *TG* triglyceride, *HDL-C* high-density lipoprotein cholesterol, *LDL-C* low-density lipoprotein cholesterol, *VLDL-C* very-low-density-lipoprotein cholesterol, *CRI* Coronary Risk Index, *AI* Atherogenic Index, *TG/HDL-C* triglyceride-to-HDL-C ratio, *SD* standard deviation, *CI* confidence interval, *q-value* corrected *p* value.

The comparison of the obesity-related anthropometric and metabolic parameters between overweight/obese and normal-weight individuals was determined by Mann–Whitney U test. The mean ± SD and mean ranks were calculated to show the difference of parameters between overweight/obese and normal-weight individuals. The analysis was corrected for multiple comparisons via the Benjamini–Hochberg method of false discovery rate (FDR) control. A *p* value < 0.05 was considered statistically significant. The significant *p* values are indicated in **bold**.

**Table 2:** Comparison of GRS between overweight/obese and normal-weight individuals.

GRS	OW/OB individuals (*n* = 306)	NW individuals (*n* = 300)	*p* value
Mean ranks	322.66	283.96	**0.005**
Minimum	2	1	
Maximum	9	9	

*GRS* genetic risk score, *OW/OB* overweight/obese, *NW* normal-weight.

The *p* value for GRS was calculated by the Mann–Whitney U test. The *p* value was considered significant and indicated in **bold** when *p* value < 0.05.

### Allele frequencies and association of individual variants with overweight/obesity

The risk allele frequencies (RAFs) of all the variants are given in Table 3. The RAFs for all the variants except rs6265 were higher in the OW/OB as compared to NW individuals; however, this difference was found to be statistically significant for the *TMEM18* rs7561317 only. Consequently, the multinomial logistic regression analysis also revealed a significant association of only rs7561317 with overweight/obesity (Table 3).

**Table 3:** Comparison of RAFs of the variants between OW/OB and NW individuals along with the association of the variants with overweight/obesity.

Gene/locus	Variant	Variant location	Risk allele	RAFs OW/OB individuals	RAFs NW individuals	OR (95% CI)	*p* value
*MC4R*	rs17782313	Intergenic	C	43.8%	40.3%	1.144 (0.911–1.437)	0.246
*BDNF*	rs6265	Missense	A	19.8%	19.8%	0.995 (0.751–1.318)	0.972
*FTO*	rs1421085	Intron	C	33.8%	30.8%	1.151 (0.902–1.471)	0.259
*TMEM18*	rs7561317	Intergenic	G	87.9%	81.8%	1.571 (1.146–2.153)	**0.005**
*NEGR1*	rs2815752	Intron	A	66.8%	64.3%	1.137 (0.899–1.483)	0.284

*OW/OB* overweight/obese, *NW* normal-weight, *RAFs* risk allele frequencies, *OR* odds ratio, *CI* confidence interval.

The data represents risk allele frequencies (RAFs) in overweight/obese and normal-weight individuals along with odds ratios and confidence intervals in parenthesis. Association was tested using multinomial logistic regression assuming an additive model. The analysis was performed by adjusting age and gender. An adjusted *p* value < 0.05 was considered significant. The significant *p* value is indicated in **bold**.

### Association of the GRS with obesity-related anthropometric parameters

A significant association of the GRS with nearly all the anthropometric variables such as body weight, BMI, WC, HC, WHR, WHtR, SFTs (biceps, triceps, supra-iliac, abdominal, thigh, and sub-scapular), and %BF was observed (Table 4). The strongest association of the GRS was seen with augmented body weight, BMI, WC, and WHtR (*p* = 0.007) followed by enhanced HC, biceps and thigh SFTs (*p* = 0.008), sub-scapular SFT (*p* = 0.018), WHR, triceps SFT and % BF (*p* = 0.02), and abdominal and supra-iliac SFTs (*p* = 0.04).

**Table 4:** Association of the genetic risk score with obesity-related anthropometric variables.

Parameters (unit of measurement)	β (S.E.)	95% CI	*p* value	*q-*value
Weight (Kg)	1.863 (0.574)	0.736–2.989	**0.001**	**0.007**
Height (cm)	− 0.230 (0.270)	− 0.760 to 0.300	0.395	0.395
BMI (Kg/m^2^)	0.691 (0.203)	0.292–1.089	**0.001**	**0.007**
WC (cm)	1.651 (0.519)	0.633–2.670	**0.002**	**0.007**
HC (cm)	1.188 (0.403)	0.396–1.980	**0.003**	**0.008**
WHR	0.006 (0.002)	0.001–0.010	**0.016**	**0.021**
WHtR	0.029 (0.009)	0.010–0.047	**0.002**	**0.007**
Biceps SFT (mm)	0.682 (0.233)	0.224–1.140	**0.004**	**0.008**
Triceps SFT (mm)	0.798 (0.332)	0.145–1.450	**0.017**	**0.021**
Sub-scapular SFT (mm)	0.879 (0.339)	0.213–1.545	**0.010**	**0.018**
Abdominal SFT (mm)	1.015 (0.481)	0.070–1.960	**0.035**	**0.041**
Thigh SFT (mm)	1.558 (0.534)	0.510–2.606	**0.004**	**0.008**
Supra-iliac SFT (mm)	0.858 (0.421)	0.031–1.685	**0.042**	**0.045**
% BF	0.689 (0.275)	0.148–1.229	**0.013**	**0.020**

*BMI* body mass index, *WC* waist circumference, *HC* hip circumference, *WHR* waist-to-hip ratio, *WHtR* waist-to-height ratio, *SFT* skinfold thickness, *% BF* body fat percentage, *SE* standard error, *CI* confidence interval, *q-value* corrected *p* value.

The association of the genetic risk score with anthropometric traits was determined by linear regression. Effect size (β) and 95% confidence intervals were computed to seek rise or fall in the selected parameter per each risk allele increase. All association analyses were adjusted for age and gender and corrected for multiple comparisons via the Benjamini–Hochberg method. A *p* value < 0.05 was considered statistically significant. The significant *p* values are indicated in **bold**.

### Association of the GRS with obesity-related metabolic traits

No significant association of the GRS with any of the obesity-related metabolic variables such as SBP, DBP, TyG, FBG, fasting insulin, HOMA-IR, TC, TG, HDL-C, LDL-C, VLDL-C, CRI, AI, and TG/HDL-C was seen (Table 5).

**Table 5:** Association of the genetic risk score with obesity-related metabolic traits.

Parameters (unit of measurement)	β (S.E.)	95% CI	*p* value	*q-*value
SBP **(**mmHg)	0.757 (0.382)	0.006–1.508	0.048	0.168
DBP **(**mmHg)	0.565 (0.278)	0.019–1.112	0.043	0.168
TyG index	− 0.030 (0.017)	− 0.063 to 0.004	0.079	0.184
FBG (mg/dL)	0.308 (0.552)	− 0.776 to 1.392	0.577	0.809
Fasting insulin (µL U/mL)	− 0.122 (0.400)	− 0.908 to 0.663	0.760	0.819
HOMA-IR	− 0.017 (0.116)	− 0.246 to 0.212	0.883	0.883
TC (mg/dL)	− 0.939 (1.131)	− 3.161 to 1.282	0.407	0.633
TG (mg/dL^−1^)	− 3.899 (1.906)	− 7.642 to − 0.156	0.041	0.168
HDL-C (mg/dL)	0.112 (0.265)	− 0.408 to 0.632	0.673	0.819
LDL-C (mg/dL)	− 1.428 (1.031)	− 3.452 to 0.595	0.166	0.326
VLDL-C (mg/dL)	− 0.746 (0.376)	− 1.485 to − 0.007	0.048	0.168
CRI	− 0.015 (0.049)	− 0.111 to 0.080	0.753	0.819
AI	− 0.045 (0.034)	− 0.112 to 0.022	0.186	0.326
TG/HDL-C	− 0.155 (0.084)	− 0.319 to 0.009	0.064	0.179

*SBP* systolic blood pressure, *DBP* diastolic blood pressure, *TyG* the product of triglycerides and glucose, *FBG* fasting blood glucose, *HOMA-IR* homeostatic model assessment-insulin resistance, *TC* total cholesterol, *TG* triglyceride, *HDL-C* high-density lipoprotein cholesterol, *LDL-C* low-density lipoprotein cholesterol, *VLDL-C* very-low-density-lipoprotein cholesterol, *CRI* Coronary Risk Index, *AI* Atherogenic Index, *TG/HDL-C* triglyceride-to-HDL-C ratio, *SE* standard error, *CI* confidence interval, *q-value* corrected *p* value.

The association of the genetic risk score with metabolic traits was determined by linear regression. Effect size (β) and 95% confidence intervals were computed to seek rise or fall in the selected parameter per each risk allele increase. All association analyses were adjusted for age and gender and corrected for multiple comparisons via the Benjamini–Hochberg method. A *p* value < 0.05 was considered statistically significant.

## Discussion

Overweight/obesity is a multifactorial metabolic disorder having a complex genetic background ^38^. In this context, the estimated heritability of obesity ranges from 40 to 70%^39^. Numerous candidate gene studies and GWAS have discovered several obesity-related genetic variants^40^, however, the additive effect of these variants on obesity risk has been reported in very few studies. In particular, the studies for exploring the combined effect of multiple genetic variants on the risk of obesity and related traits regarding Pakistani population are in their infancy. As common obesity has a polygenic inheritance, the assessment of cumulative effect based on GRS is very crucial to fully comprehend the components of genetic architecture and physiopathology of obesity. The striking potential of GRS for identifying individuals at a higher risk of developing overweight/obesity has been revealed in this regard^13, 14^.

The rs1421085 of the *FTO* gene (T > C) is anticipated as the causal variant for overweight/obesity and it has been linked with a higher total energy intake and more eating episodes per day^41, 42^. The obesity-increasing effect of *FTO* rs1421085 has been shown to disrupt ARID5B-mediated repression of *IRX3* and *IRX5* expression in pre-adipocytes that in turn leads to the excessive accumulation of triglycerides, increased adipocytes size, reduced mitochondrial oxidative capacity, and reduced white adipocytes browning, resulting in reduced mitochondrial thermogenesis^43^. The variant rs17782313 (T > C) located 188 kb downstream of the *MC4R* gene is strongly linked with obesity and the disruption of the transcriptional control of *MC4R* has been proposed as the likely mechanism of this variant^44^. Likewise, the rs6265 variant (G > A) in the coding region of the *BDNF* gene that involves Valine to Methionine substitution at the 66th amino acid position (Val66Met) of the N-terminal domain of pro-BDNF has also been linked with obesity. The intracellular trafficking and depolarization-induced release of BDNF are hampered due to this risk variant (rs6265)^45, 46^. The rs7561317, located about 22 kb downstream of *TMEM18* is also an obesity-associated genetic variant that may diminish the expression of the *TMEM18* gene and thus play a role in the manifestation of obesity^33, 47^. The rs2815752 variant (G > A) positioned 20 kb upstream of the *NEGR1* gene presumably decreasing its expression is linked with increased risk of obesity^48^.

The current study computes the GRS based on the above-discussed five obesity-linked key loci (*FTO* rs1421085, *MC4R* rs17782313, *BDNF* rs6265, *TMEM18* rs7561317, and *NEGR1* rs2815752), which were previously found to be individually associated with obesity and related anthropometric and metabolic measures in the same sample of Pakistani population^23–26^. In our previous studies, the aforementioned individual associations were sought using multiple genetic models and in the case of obtaining association in more than one model, the *h*-index was calculated to indicate the relevant mode of inheritance (such as dominant, recessive, or over-dominant). Also, associations of the two variants *FTO* rs1421085^24^ and *TMEM18* rs7561317^25^ were obtained without gender stratification while those of the other two namely *MC4R* rs17782313^23^ and *NEGR1* rs2815752^26^ were observed after gender stratification in females only. Moreover, no association was found for *BDNF* rs6265^49^. However, in the current study, we used a single additive model for simultaneously encompassing all the aforementioned five different genetic variants in the analysis to compute GRS for obesity-related anthropometric and metabolic phenotypes. Moreover, the sample population was not stratified based on gender in the current study. Therefore, the use of an additive model and non-stratification of the sample population might be the possible reasons that only rs7561317 was found to be associated with the overweight/obesity, when before computing GRS, individual associations of the aforementioned variants with obesity were sought in the current study.

The current study has been undertaken to determine the susceptibility when the combined effects of the above-mentioned five variants are considered because common obesity has a polygenic inheritance as indicated before. Moreover, the cumulative effect of these five variants for the increased risk of obesity has not been studied before in the Pakistani population. In the present study, the GRS based on the aforementioned five genetic variants appeared to be significantly associated with the obesity-related anthropometric parameters such as body weight, BMI, WC, HC, WHR, WHtR, SFTs, and BF% in a sample of Pakistanis regardless of age and gender. This indicates that individuals with high GRS may be more predisposed to the development of overweight/obesity. It must be noted that four out of five genetic variants could not exhibit any significant association with overweight or obesity when associations for individual variants were sought in the current study. However, the significant association of GRS based on a count of risk-associated alleles across a panel of five aforementioned genetic variants was observed, which point to the benefit of identifying a significant association based on the additive count of multiple genetic variants that individually may or may not appear to exhibit a significant association with the risk of overweight/obesity in a sample population. This implies that the use of GRS is comparatively a better approach to estimate the genetic risk of overweight/obesity based on different multiple common risk variants rather than individual risk variants especially when the sample size is not very large.

Previously, two studies also reported a significant association of GRS based on obesity-associated genetic variants with the increased risk of being obese in Pakistanis^50, 51^. However, the GRS in these aforementioned two studies were based on different sets of genetic variants, which differed not only between these studies but also differed with our study. This indicated the additive power of GRS over an individual variant because common obesity has a polygenic inheritance implying that various obesity-associated genetic variants act in concert to modulate the bodyweight^52^. Our and the two aforementioned studies^50, 51^ can be considered to create a panel of genetic variants for estimating the risk of an individual for developing obesity. The association of the GRS with almost all anthropometric traits (body weight, BMI, WC, HC, WHR, WHtR, skinfold thicknesses, and %BF) in our study showed the additive effect of these variants on fat distribution resulting in increased body weight. It is important to note that WC and WHtR are the main determinants of abdominal obesity, cardiovascular diseases, and increased mortality risk^53–56^. Thus, the higher GRS (constructed in our study) may escalate the risk of cardiovascular disease and mortality via increasing WC and WHtR.

An interesting and important aspect of the current study is that we not only computed and sought the association of the GRS with the obesity-related anthropometric measures but also with obesity-related metabolic measures. Nevertheless, the GRS in the current study showed a lack of association with all metabolic traits including the parameters related to glucose and lipid metabolism, and blood pressure. Likewise, none of the aforementioned five genetic variants in our previous studies individually showed a significant association with any of the obesity-related metabolic traits in the same sample of the Pakistani population^23–26, 49^. However, it must be noted that the values of nearly all the metabolic parameters (like anthropometric variables) were found significantly higher in overweight/obese individuals as compared to normal-weight individuals. Moreover, the GRS computed in our study was based on the genetic variants mainly involved in energy homeostasis and body weight regulation via regulating appetite and energy expenditure as already mentioned in the introduction section. Thus, it can be said that these variants may indirectly influence metabolism via increasing BMI and other obesity-related anthropometric measures.

In the end, it can be concluded that the cumulative or combined effect based on GRS may be valuable for better comprehension of the genetic susceptibility to obesity and may help researchers to better understand trait biology.

## Material and methods

### Study design, setting, and ethics

It was an analytical observational study based on a case–control design and was performed at Dr. Panjwani Center for Molecular Medicine and Drug Research (PCMD), International Center for Chemical and Biological Sciences (ICCBS), University of Karachi (UoK), Pakistan. All procedures performed in the study involving human participants were in accordance with the ethical standards of the Independent Ethics Committee (IEC) of the ICCBS and Advanced Studies and Research Board (ASRB) of the University of Karachi, Pakistan, and with the 1964 Helsinki Declaration and its later amendments or comparable ethical standards. The study was approved by the Independent Ethics Committee (IEC) of the ICCBS and Advanced Studies and Research Board (ASRB) of the University of Karachi.

### Sample population and phenotypic traits

The study involved a total of 606 participants including 306 overweight/obese (OW/OB) and 300 normal-weight (NW) individuals. The participants were recruited from the general population of Karachi, a metropolitan city of Pakistan, including universities and colleges of the city using the simple random sampling without replacement technique. However, all the participants were not permanent residents of the city. All the participants or their parents/guardians (in the case of children or adolescents) signed the written informed consent before participation in the study. The demographic information regarding the recruited participants was acquired through a questionnaire. The body mass index (BMI) reference ranges for adults were considered according to World Health Organization (WHO), whereas for children/adolescents, BMI reference ranges were considered according to growth charts of the Center for Disease Control and Prevention (CDC). However, the individuals with a history of taking drugs such as antidepressants, phenothiazine, and steroids, etc., and having any type of endocrinological disorder were not included in the study. Blood pressure (BP) was measured from the right-upper arm according to the standard procedure by using a mercury column sphygmomanometer (Certeza medical, Germany) while the individual was sitting comfortably. The BP of each individual was measured twice to calculate the average value. The body weight was measured in kilograms (kg) with the precision of up to 0.1 kg by using a mechanical weighing balance (Seca 755, Germany). Moreover, the body height was measured in centimeters (cm) with the precision of up to 0.1 cm by using a portable stadiometer (Seca 214, Germany). Both measurements were assessed without shoes and in light clothes. To calculate BMI, height was converted into meters (m) and then the value of weight (kg) was divided by the value of height in the square (m^2^). The waist circumference (WC) and hip circumference (HC) were measured with a non-elastic measuring tape. The skinfold thicknesses (SFTs) such as biceps, triceps, suprailiac, abdomen, thigh, and sub-scapular were measured in millimeters (mm) by using a skin-fold caliper (Slim Guide, MI, USA). All the measurements were taken thrice to compute the average value for each SFT. The waist-to-hip ratio (WHR) and waist-to-height ratio (WHtR) were calculated using the values of WC, HC, and height. The body fat percentage (%BF) was computed using the sum of SFTs by employing gender-specific formulae^57^. The fasting (8–12 h) blood samples from each participant were collected in two separate vacutainer tubes for subsequent serum and DNA isolation. Fasting blood glucose was measured using a glucose monitoring system (Abbott, UK) while fasting insulin levels were measured by ELISA (enzyme-linked immunosorbent assay) using a commercial kit (DIA source, Belgium). The values of fasting glucose and insulin were used to calculate homeostatic model assessment-insulin resistance (HOMA-IR) by applying relevant formula^58^. Moreover, lipid parameters (total cholesterol, triglycerides, HDL-C, and LDL-C) were determined on a chemistry analyzer (Hitachi 902, Japan) by consuming commercial kits (Merck, Germany) based on enzymatic endpoint assays. The values of triglycerides were divided by 5 to calculate VLDL-C. Also, coronary risk index (CRI), atherogenic index (AI), triglyceride-to-HDL-C ratio (TG/HDL-C) were calculated^59^. Furthermore, the product of triglyceride and glucose (TyG) index was also computed^60^.

### DNA extraction and genotyping

DNA was extracted from the collected blood samples by the spin column method consuming commercially available kits (Bio Basic, Canada). The genetic variants (*MC4R* rs17782313, *BDNF* rs6265, *FTO* rs1421085, *TMEM18* rs7561317, and *NEGR1* rs2815752) were genotyped using TaqMan allelic discrimination assays (assay ID of the rs17782313: C__32 667060_10, cat. # 4351376, ABI, Foster City, CA, USA; assay ID of the rs6265: C__11592758_10, cat. # 4351379, ABI, Foster City, CA, USA; assay ID of the rs1421085: C__8917103_10, cat. # 4351379, ABI, Foster City, CA, USA; assay ID of the rs7561317: C__32 667 060_10, cat # 4351376, ABI, Foster City, CA, USA; assay ID of rs2815752: C__26668839_10, cat # 4351379, ABI, Foster City, CA) containing specific primers and probes and TaqMan master mix (Applied Biosystems, USA) on a real-time PCR System (ABI 7500, USA). Every batch of PCR experiments consisted of positive control for each genotype of all the variants as well as two negative controls (no template controls). 20% of the samples were genotyped twice for reproducibility.

### Statistical analysis

All statistical analyses were performed utilizing the Statistical Package for Social Sciences version 21 (SPSS, IBM statistics). Hardy–Weinberg Equilibrium (HWE) test was applied to determine whether the genotypic distributions of all the variants were in HWE. The Shapiro–Wilk test was availed to check the normality of the data. The obesity-associated risk alleles of *MC4R* rs17782313, *BDNF* rs6265, *FTO* rs1421085, *TMEM18* rs7561317, and *NEGR1* rs2815752 were C, A, C, G, and A, respectively. The homozygous wild type (non-risk), heterozygous (risk), and homozygous mutant (risk) genotypes were coded as 0, 1, and 2, respectively. The GRS was constructed by summing the number of risk alleles (0, 1, and 2) of the aforesaid genetic variants. Since we included five bi-allelic variants, an individual may have a minimum of 0 and a maximum of 10 risk alleles. However, none of the individuals was found to have either 0 or 10 risk alleles. The study individuals were found to have a minimum of 1 and a maximum of 9 risk alleles. The GRS and the other continuous variables were compared between overweight/obese and normal-weight individuals by employing Mann–Whitney U test. Allelic frequencies of all the variants were calculated by direct count. The association of each variant with overweight/obesity was sought by assuming an additive model. Multinomial logistic regression was applied and the odds ratio (OR) along with 95% confidence intervals (CI) was calculated to predict the overweight/obesity risk associated with each variant as well as with GRS. The association of GRS with obesity-related anthropometric and metabolic parameters was determined by linear regression. For conformity to the linearity assumption of the linear regression, all non-normal data were transformed into normal distribution through a rank-based inverse normal transformation. The effect sizes (β) along with 95% CI were determined for all anthropometric and metabolic parameters. All analyses were adjusted for confounders such as age and gender and also corrected for multiple comparisons by the Benjamin-Hochberg method as per requirement^61^. A *p* value < 0.05 was considered statistically significant for all analyses.

## Data Availability

All data generated or analyzed during this study are included in this published article.
